# A Digital Music-Based Mindfulness Intervention for Black Americans With Elevated Race-Based Anxiety: A Multiple-Baseline Pilot Study

**DOI:** 10.2196/49284

**Published:** 2023-08-16

**Authors:** Grant Jones, Felipe Herrmann, Matthew K Nock

**Affiliations:** 1 Harvard University Cambridge, MA United States

**Keywords:** Black music, mindfulness, meditation, single-case experiment, race, anxiety, mindfulness, digital health intervention, low income, Black community, racial disparity

## Abstract

**Background:**

Race-based anxiety is a substantial health issue for the Black community. Although mindfulness interventions have demonstrated efficacy for alleviating anxiety, three central barriers prevent Black Americans from accessing existing mindfulness treatments: high costs, excessive time commitments, and limited cultural relevance. There is a need for novel mindfulness interventions for the Black community that can overcome these barriers.

**Objective:**

The goal of this web-based study was to examine the preliminary efficacy, feasibility, and acceptability of a novel digital music-based mindfulness intervention for middle-to-low-income Black Americans with elevated race-based anxiety.

**Methods:**

This study used a nonconcurrent multiple-baseline design (n=5). The intervention featured contributions from Lama Rod Owens (a world-renowned meditation teacher and *LA Times* best-selling author) and Terry Edmonds (the former chief speechwriter for President Bill Clinton). We examined the effect of the intervention on state anxiety and assessed its feasibility and acceptability using quantitative and qualitative measures.

**Results:**

Results revealed that administration of the intervention led to significant decreases in state anxiety (Tau-U range –0.75 to –0.38; *P* values<.001). Virtually all feasibility and acceptability metrics were high (ie, the average likelihood of recommending the intervention was 98 out of 100).

**Conclusions:**

This study offers preliminary evidence that a digital music-based mindfulness intervention can decrease race-based anxiety in Black Americans. Future research is needed to replicate these results, test whether the intervention can elicit lasting changes in anxiety, assess mechanisms of change, and explore the efficacy of the intervention in real-world contexts.

## Introduction

Anxiety is a substantial health issue within the Black community. Black Americans endure greater lifetime exposure to stressors and adversity than other racial or ethnic groups in the United States [1]. Accordingly, anxiety disorders among Black Americans are more chronic, functionally impairing, and treatment resistant than those among White Americans [2]. Furthermore, health care inequities limit opportunities for Black Americans to address their anxiety via mental health services [3,4]. In addition, race-based discrimination is a unique predictor of anxiety disorders within the Black community [5,6]. In recent years, increases in racism have been associated with a disproportionate increase in self-reported anxiety among Black Americans [7,8]. Yet to our knowledge, only four studies have examined interventions for race-based anxiety in Black Americans, and all of these studies have been preliminary or targeted niche samples (ie, caregivers, veterans, and women at risk for cardiovascular disease) [9-12]. Additional research is needed to develop and test scalable interventions to address anxiety in the Black community, particularly anxiety that comes from racism and discrimination.

Interventions that promote mindfulness—a nonjudgmental and conscious attention to the present moment—represent a promising source of support for Black Americans. Mindfulness interventions have demonstrated utility in alleviating symptoms of stress and anxiety in various systematic reviews and meta-analyses [13-15]. Additionally, within Black Americans, higher levels of mindfulness may act as a protective moderator of the relationship between racial discrimination and anxiety symptoms [16,17]. Hence, interventions that promote mindfulness may help reduce anxiety among Black Americans.

Despite the potential utility of mindfulness treatments, 3 main obstacles often impede Black Americans from receiving evidence-based mindfulness-based interventions. First, most mindfulness interventions are not culturally tailored for Black Americans. A 2018 systematic review examined cultural adaptations and diversity considerations within mindfulness meditation-based intervention studies; out of 24 mindfulness meditation-based intervention diversity-focused studies, just 3 were culturally adapted to a specific racial or ethnic group, and only a single mindfulness intervention was tailored specifically for Black Americans [18]. To our knowledge, only a handful of such studies have been published since [11,19,20]. Many within the mindfulness community believe that culturally tailored interventions can increase effectiveness and acceptability in diverse populations [18,21,22], and Black American focus groups have suggested incorporating culturally familiar elements (eg, Black spirituality, culturally familiar terminology, and Black facilitators) when administering mindfulness interventions [23,24]. Therefore, culturally relevant mindfulness interventions may increase the acceptability of mindfulness treatments for the Black community.

Second, accessing evidence-based mindfulness-based resources can be expensive [25]. For instance, a single mindfulness-based stress reduction course costs US $540 on average and can cost US $1533 [26]. More cost-effective mindfulness interventions are needed to reach Black Americans, particularly those at lower income levels who cannot afford existing treatments.

Third, mindfulness interventions often require excessive in-person time commitments; mindfulness-based stress reduction requires 8 weekly two-and-a-half-hour sessions, with an additional 6-hour retreat. Many Black Americans cannot afford to spend time seeking and accessing treatments, particularly those from lower-income backgrounds with heavy work and family demands [27]. Hence, there is a critical need to develop and disseminate culturally relevant, cost-effective, and time-limited mindfulness interventions to underserved populations.

A digital music-based mindfulness intervention may represent a promising treatment avenue for Black Americans and can address the aforementioned barriers. First, music represents a potential avenue for delivering culturally relevant mindfulness to the Black community. In a study examining perceptions of mindfulness among low-income Black Americans, participants noted mindfulness states to be similar to those elicited by worship music [22]. A study examining the effects of classical music on Black Americans found that 3 weeks of daily listening significantly increased mindfulness while attenuating symptoms of stress and anxiety [28]. Furthermore, a preliminary 2020 study demonstrated that a precomposed web-based music intervention not only decreased anxiety but also increased mindfulness [29]. Second, there is suggestive evidence that brief web-based mindfulness interventions—as short as 15 minutes—can reduce stress and anxiety [30-33]. Third, disseminating a digital music-based intervention via web-based music streaming platforms can also provide low-cost access to the intervention while simultaneously reducing excessive in-person time demands. Thus, a digital music-based mindfulness intervention designed specifically for Black Americans may be able to elicit strong reductions in race-based anxiety with high feasibility and acceptability ratings.

This study aims to examine the feasibility and preliminary efficacy of a novel digital music-based mindfulness intervention for middle-to-low-income Black Americans with elevated race-based anxiety. We aimed to test the intervention in this income bracket as middle-to-low-income Black people face additional barriers to accessing mindfulness interventions than do individuals from higher income brackets; thus, our investigation would be able to provide evidence for the feasibility of the intervention in a subset of the Black population that is in particular need of this treatment. Furthermore, our study was designed to test whether this intervention leads to immediate decreases in state anxiety (ie, momentary anxiety). If so, future studies would test whether the intervention causes lasting reductions in trait anxiety (ie, persistent anxiety).

We used a nonconcurrent multiple baseline design, which entails taking repeated measures across time to compare an outcome of interest between 2 phases: an initial baseline control period (no intervention) and a subsequent intervention period during which the intervention is administered [34]. Furthermore, nonconcurrent designs also entail randomizing individuals to baseline conditions of varying lengths, aiding with the assessment of the comparison of the intervention and baseline phases. If changes in one’s outcomes occur when a treatment is applied in a nonconcurrent design, it becomes easier to infer that such a change was caused by the treatment and not simply by the passage of time (as it would be unlikely that a given outcome would randomly and markedly change for all participants exactly at the end of baseline periods of varying lengths).

Generally, multiple-baseline designs allow for preliminary causal inferences using relatively small sample sizes [35]. This approach is advantageous for testing novel interventions in populations that are challenging to recruit into studies, such as racial minority groups (eg, Black Americans). This investigation has the potential to elucidate a novel avenue for alleviating anxiety among Black Americans.

## Methods

### Overview

The intervention was designed by the lead author and consists of guided meditations, songs, and poems totaling 25 minutes in duration. All elements of the intervention were set to background music tracks, and all music production and composition were informed by Black diasporic music traditions such as rhythm and blues, soul, jazz, and Black alternative music. Furthermore, music composition and production were informed by stress and anxiety reduction principles from the music therapy literature, such as creating music that has a slower tempo [36,37] and features sounds from nature [38-40]. Table 1 provides an overview of the tracks, their order in the intervention, the original authors, the content type, and the meditation practice themes explored.

**Table 1:** An overview of the content within the intervention, the original authors, and the meditation practice themes explored within each component of the intervention.

Track name	Original authors	Content type	Meditation practice themes explored	Length (min)
Where are you?	Lama Rod Owens and Grant Jones	Guided meditation	Body awareness; healing from racism; acceptance of difficult emotions	5
May I be enough.	Grant Jones	Song	Self-compassion; self-acceptance	3
Question marks	Terry Edmonds	Poem	Self-acceptance	1
Slow down.	Grant Jones	Song	Present-moment awareness	4
Dark voices	Terry Edmonds	Poem	Black spirituality; belonging	2
When will you awaken?	Lama Rod Owens and Grant Jones	Guided meditation	Body awareness; healing from racism; breath awareness; thought awareness	5
Truly happy	Grant Jones	Song	Radical joy (ie, finding joy in difficult circumstances)	4

### Guided Meditations

Guided meditations were drafted and delivered by Lama Rod Owens, a Black, queer meditation teacher who is formally ordained within the Kagyu School of Tibetan Buddhism after completing a 3-year silent meditation retreat. He is a world-renowned meditation teacher and *LA Times* best-selling author who writes and teaches about the intersection of meditation, race, social justice, identity, and liberation. Accordingly, Lama Rod drafted meditations specifically for this intervention that were designed to explore these topics (ie, “...let go of how systems of power, violence, and dominance have defined you” from “When will you awaken?”). The lead author provided revisions and edits to these meditations ahead of their inclusion in the intervention.

### Songs

Songs were composed, performed, and produced by the lead author. The songs featured singing, instrumentation, and lyrics that explicitly referenced mindfulness practices such as self-compassion (ie, “May I be enough as is...” from “May I be enough.”) and the acceptance of difficult emotions (ie, “...face the things we hide, reach out, embrace it...” from “Slow down”).

### Poetry

Poetry for this intervention was written and delivered by Terry Edmonds, the first Black person to serve as a speechwriter for a US president and the former chief speechwriter for President Bill Clinton. His poetry explores themes related to Black spirituality, Black community and belonging, and radical self-acceptance (ie, “Do I have the courage to be, the unimaginable me?...Can I make peace?” from “Question Marks”). Terry and the lead author worked together to select poems that Terry had previously drafted to include in the intervention.

### Participants

Participants were recruited from Prolific, a web-based study participant recruitment platform. We screened and recruited participants into our study in a 2-step process. We used prescreening criteria on Prolific to initially recruit only middle-to-low-income Black Americans (<US $50,000/yr) who endorsed elevated anxiety as well as familiarity with meditation. Next, we recruited 60 of these potential participants to take a screener survey, after which we recruited individuals who endorsed using music as a form of emotional support. For this initial study, we selected only individuals who were familiar with meditation and used music as a form of emotional support, as these individuals would be able to provide an informed initial assessment of the viability of our novel approach (ie, if the intervention fails to demonstrate feasibility or preliminary efficacy in a population that is well-positioned to evaluate and experience its intended impacts, it would suggest a low likelihood that our intervention effects would generalize to individuals who are unfamiliar with meditation and indicate the need for major revisions to our approach).

**Figure 1:**
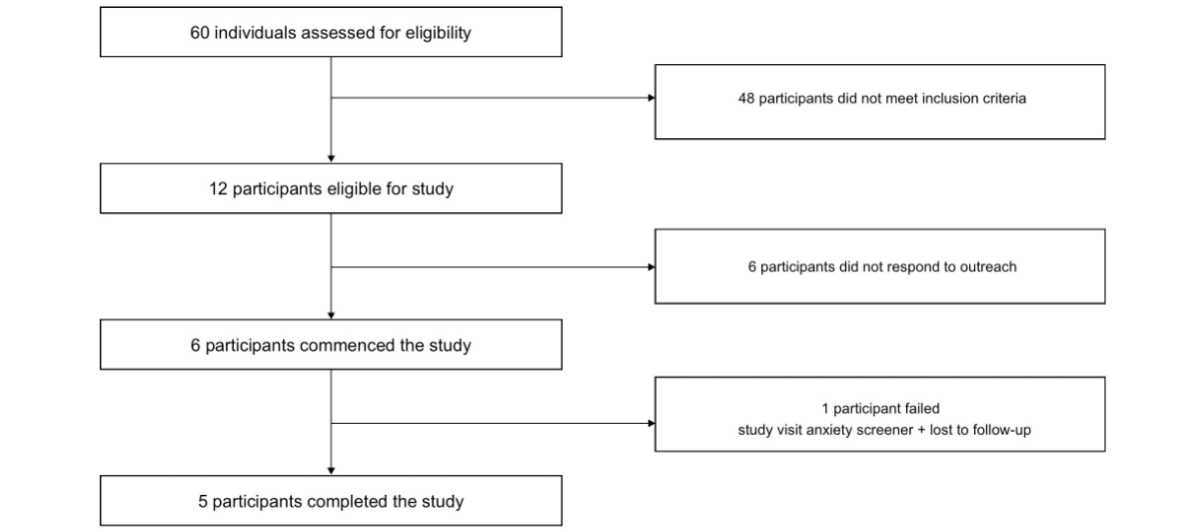
CONSORT (Consolidated Standards of Reporting Trials) diagram for our study.

Finally, we used the screener to recruit individuals who endorsed that the majority of their anxiety comes from racism and/or discrimination (≥60 on a scale of 0 [not at all] to 100 [entirely] for the following question: “If you are a racial or ethnic minority, how much do you feel that racism and/or discrimination contribute to your elevated anxiety levels?”). All participants who completed the screener received US $1, regardless of their eligibility. We contacted eligible participants to participate in the study, and interested participants signed up for a virtual study visit using Calendly, a web-based scheduling tool. Of the 60 individuals who completed the screener, 12 were eligible for our study. Six individuals scheduled and commenced a study visit, and the other 6 eligible individuals did not respond to our outreach to set up a study visit. Overall, 5 individuals completed our study protocol, as 1 participant failed our screener and declined to reschedule his visit. Figure 1 presents a CONSORT (Consolidated Standards of Reporting Trials) diagram that illustrates the flow of participants through our screening and enrollment process.

### Ethics Approval

This study was conducted by a research team based at Harvard University, and all materials and procedures for this study were approved by the Harvard Institutional Review Board (protocol IRB21-0256).

### Study Protocol

Study visits took place on Zoom, and participants were required to be on video for the entire study visit. The videoconference format allowed us to confirm the identity of participants, ensure that participants were not bots (bots can greatly damage the fidelity of web-based studies), and monitor participant engagement with the intervention. Eligible participants were instructed to book their study visits during a time when they were experiencing elevated anxiety. On the day of the study visit, participants were screened for their state anxiety, and any participants who scored below 14 (out of 24) on the State-Trait Anxiety Inventory-6 (STAI-6) scale were informed that they did not pass the screener form and were given the option to reschedule their study visit for another time. Two participants failed this screener; one opted out of the study, and the other commenced his study visit at a later time that day.

The study visit had 2 main phases, during which we assessed state anxiety levels every 2 minutes. The first phase was the baseline phase (A phase). Participants were randomized to 1 of 5 baseline conditions that varied in length (10, 14, 18, 22, or 26 minutes of baseline; 5-13 baseline measurement periods). During baseline, participants were instructed to go about their business as usual, which could involve surfing the web, grabbing a snack, destressing, or engaging in any other activities of their choosing. The second phase was the intervention phase (B phase), during which we administered the music-based mindfulness intervention while continuing to assess anxiety. Participants were invited to find a comfortable position, attend to the intervention, and pay attention to any directions they received within the intervention. No other instructions were given. As the intervention totaled 25 minutes and we assessed anxiety every 2 minutes, there were 12 intervention assessments for each participant.

Following the baseline and intervention phases, we also assessed the feasibility and acceptability of the intervention with a series of quantitative and qualitative questions.

At the end of the study, participants were debriefed about our hypotheses and paid US $60 for their participation.

### Measures

#### State Anxiety

We used the STAI-6 (State-Trait Anxiety Inventory-6) to measure state anxiety within this study; this scale is a short form of the Spielberger STAI-State version, a widely used scale to measure anxiety [41]. Answers for this 6-item Likert scale ranged from 1 (Not at all) to 4 (Very much so); total scores range from 6 to 24, and higher scores indicate higher levels of state anxiety.

#### Feasibility and Acceptability

Individuals were asked a series of study-specific questions about the feasibility and acceptability of the intervention. Specifically, individuals were asked:

1. How likely would you be to recommend this intervention to someone you care about from the Black community that is dealing with acute stress/anxiety? (Variable: “Likely to recommend”; 0: Not at all likely; 50: moderately likely; 100: Definitely likely)
2. To what extent was the intervention harmful or helpful for your stress/anxiety? (“Helpful for Anxiety”; 0: harmful; 50: no impact; 100: incredibly helpful)
3. To what extent did you find it difficult or easy to engage with the intervention? How practical and logistically easeful did it feel? (“Easy to Engage With”; 0: extremely difficult; 50: neutral; 100: as easy as I could imagine)
4. To what extent do you feel like the study changed your ability to be present with your thoughts and emotions? (“Improved Ability to be Present”; 0: much less able to be present; 50: no change; 100: much more able to be present)
5. To what extent do you think the study changed the way you feel about yourself? (“Improved Relationship with Self”; 0: much worse about myself; 50: no change; 100: much better about relationship to self)
6. To what extent do you feel that this intervention was made for people like you? (“Made for You”; 0: not at all; 50: somewhat; 100: exactly for me)
7. How helpful/harmful do you think it would be to experience and work with this intervention over a longer period of time (ie, daily for 1-2 weeks)? (“Longer Engagement Helpful”; 0: very harmful; 50: no difference; 100: very helpful).

### Analyses

We conducted all analyses in R version 4.1.2 using the Scan package, which is designed to conduct analyses for multiple baseline studies and other types of single-case experiments [42].

#### Tau-U Analysis

We used Tau-U analysis to assess the effect of the intervention (independent variable) on state anxiety levels (dependent variable). Tau-U analysis is one of the most widely used analytic approaches for multiple baseline studies [43].

This method assesses the nonoverlap of data between the 2 study phases (baseline vs intervention) to determine the effect of the intervention on one’s outcome of interest; additionally, this method allows one to incorporate data trends from each phase into one’s analyses and ultimately account for the effect of time when interpreting results. This approach is thus particularly advantageous for this study; state anxiety is transient, and we, therefore, expected anxiety levels to fall naturally over the course of the study. These analyses allowed us to factor in any such trends when comparing the 2 study phases.

Furthermore, although multiple baseline studies have historically been assessed using visual analyses (ie, visually examining graphs of one’s data to compare changes across study phases), these approaches are gaining increased criticism due to the subjectivity and rating inconsistencies that often accompany them [44-46]. Conversely, Tau-U analysis offers an inherently standardized, quantitative method for assessing single-case data and thus addresses a key limitation of the aforementioned visual approach.

Each Tau-U model returns 4 statistical tests:

1. A versus B (baseline vs intervention)
2. A versus B – trend A (baseline vs intervention, controlling for the trend in the baseline phase)
3. A versus B + trend B (baseline vs intervention, controlling for the trend in the intervention phase)
4. A versus B + trend B – trend A (baseline vs intervention, controlling for the baseline and intervention phase trends)

Our main outcome was an overall Tau-U model that combined the results of individual Tau-U models for each of the 5 participants. “Scan” combines these models by weighting all Tau-U values for each participant by their respective standard errors and subsequently averaging these terms. As there are various approaches to conducting Tau-U analyses, we used the methodology described in Parker et al [43], as this paper is one of the most widely cited articles on Tau-U analyses. Tau-U values typically range from –1 to 1, and the magnitude of the Tau-U value serves as a proxy for the degree of nonoverlap between 2 study phases. Furthermore, as previously stated, these values can also account for trends in the baseline and intervention phases when assessing data nonoverlap. In the case of our study, a positive Tau-U value would indicate an increase in anxiety due to the intervention, whereas a negative Tau-U value would indicate a reduction in anxiety.

#### Power Analyses

We also used the Scan package to conduct power analyses for this study. We determined that we needed at least three participants and at least five baseline measurements per participant (with 12 intervention measurements) to detect a moderate intervention effect (*d=*0.5) with requisite power (80%) and an alpha error rate below 5%. Therefore, our study design, featuring 5 participants and between 5 and 13 baseline periods per participant, was sufficiently powered. The results of our power analyses are presented in Table S1 in Multimedia Appendix 1.

## Results

### Overview

An overview of the demographic characteristics of our participants is presented in Table 2. Table 2 also provides information on the level of familiarity that participants have with meditation (variable: “Meditation Familiarity”), the frequency with which participants turn to music for emotional support (“Music for Support”), trait anxiety levels, and the extent to which racism contributes to their anxiety.

The main study results from our overall Tau-U model are presented in Table 3. Results revealed that the intervention led to a significant reduction in anxiety. Overall, all Tau-U values in the overall model—including those that adjust for the trends within the baseline and intervention phases—were negative and significant (*P*<.001), indicating that the intervention significantly reduced state anxiety.

**Figure 2:**
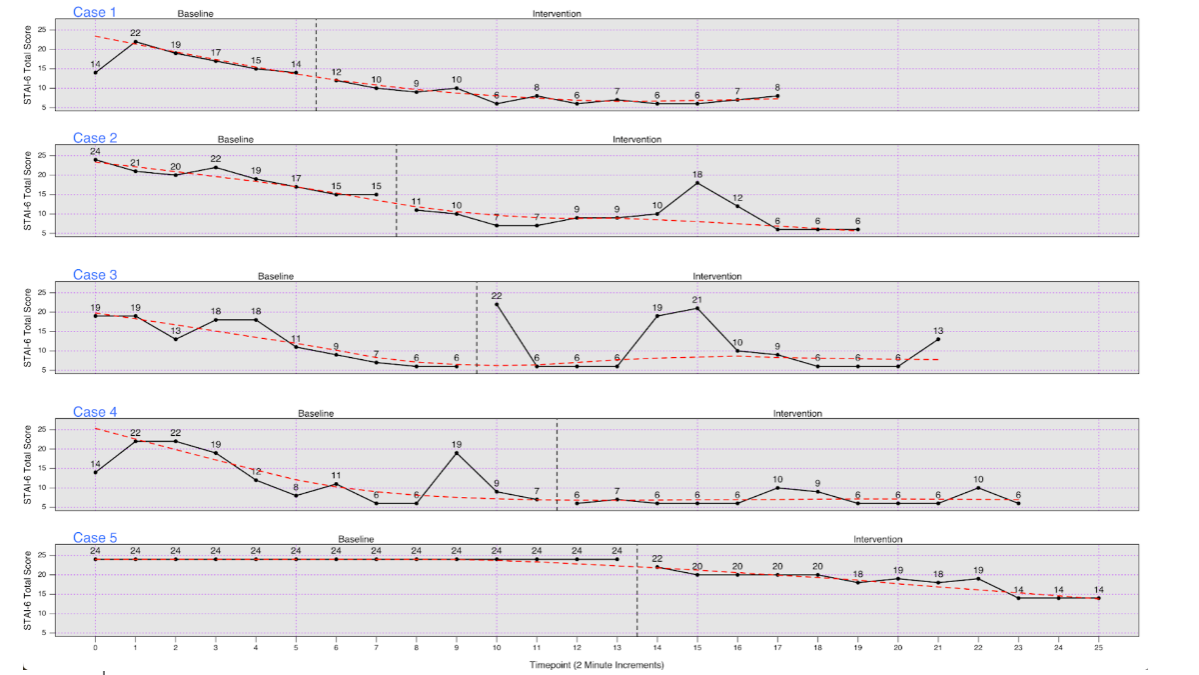
Anxiety scores STAI-6 for each of the 5 participants for each measurement period of the study. STAI-6: State-Trait Anxiety Inventory-6.

Individual-level anxiety scores for each participant at each time point are presented in Figure 2, and Tau-U results for each participant are presented in Table S2 in Multimedia Appendix 1. For 3 of 5 participants (cases 1, 2, and 5), there was a change (decrease) in level of anxiety at the start of the intervention phase, and all Tau-U values were negative and significant. For an additional participant (case 4), anxiety scores were generally much lower during the intervention than during the baseline phase, and the unadjusted Tau-U analyses (A vs B), as well as those that correct for a trend in the intervention phase (A vs B + trend B), were statistically significant. Case 3 showed weaker effects of the intervention.

**Table 2:** Sample demographics.

Participant	Gender	Age (years)	Income	Education	Meditation familiarity	Music for support	Trait anxiety (STAIT5^a^)	Racism as a cause of anxiety (%)
Case 1	Nonbinary	32	US $15,000-US $24,999	Some college (left early)	Moderately so (“I meditate on occasion”)	Often	13	60
Case 2	Male	26	US $35,000-US $49,999	Bachelor’s degree	Somewhat (“I’ve meditated once or twice”)	All the time	20	80
Case 3	Female	50	US $35,000-US $49,999	Bachelor’s degree	Moderately so (“I meditate on occasion”)	Sometimes	15	70
Case 4	Male	44	US $35,000-US $49,999	Graduate degree	Very much so (“I have a regular meditation practice”)	All the time	10	60
Case 5	Male	40	US $25,000-US $34,999	Some college (left early)	Moderately so (“I meditate on occasion”)	All the time	12	90

^a^STAIT5: State-Trait Anxiety Inventory-Trait–5.

**Table 3:** Overall Tau-U results.

Model	Tau	SE	CI	z	*P* value
A vs B	–0.75	0.11	–0.97 to –0.52	–6.54	<.001
A vs B – trend A	–0.57	0.13	–0.83 to –0.31	–4.23	<.001
A vs B + trend B	–0.58	0.09	–0.75 to –0.41	–6.80	<.001
A vs B + trend B – trend A	–0.38	0.07	–0.51 to –0.25	–5.54	<.001

### Feasibility and Acceptability Results

All participants reported that the intervention was both feasible and acceptable (Table 4). The average “Likely to Recommend” score was 98 out of 100, indicating very high acceptability for the intervention. Virtually all of the other feasibility and acceptability questions had high feasibility scores as well: “Helpful for Anxiety” (90/100), “Easy to Engage With” (89/100), “Improved Ability to be Present” (90/100), “Made for You” (93/100), and “Longer Engagement Helpful” (93/100). “Improved Relationship with Self” had moderate acceptability scores (72/100).

**Table 4:** Feasibility and acceptability results. All scores were out of 100.

Participant	Likely to recommend	Helpful for anxiety	Easy to engage with	Improved ability to be present with emotions	Improved relationship with self	Made for you	Longer engagement helpful
Case 1	100	90	75	80	60	85	80
Case 2	100	100	100	100	100	100	100
Case 3	100	100	100	100	50	100	100
Case 4	100	90	97	99	100	100	100
Case 5	89	71	71	72	50	80	86
Mean	98	90	89	90	72	93	93

### Likes, Dislikes, and Suggestions for Improvement

Participants also provided information about the number of days they believed one would need to engage with the intervention for lasting benefit (“# of Days for Lasting Benefit”), as well as their likes and dislikes or suggestions for improvement for the intervention (Table 5). The mode for “# of Days for Lasting Benefit” was 7 days and the range was between 7 days and 186 days. Likes included the quality of the music and poetry (n*=*2) and the cultural relevance of the intervention (n*=*2). The main suggestion for improvement was to include more elements of the intervention that were equipped to hold some of the more difficult emotions that were brought up by the music (n*=*2). An additional dislike or suggestion for improvement for 1 participant was to address some audio issues caused by technology in the study.

**Table 5:** Days for lasting benefit, likes, dislikes.

Participants	Days of lasting benefit, n	Likes	Dislikes or suggestions for improvement
Case 1	7	Music quality and cultural relevance	Provide more offerings to hold the emotions brought up by the music
Case 2	7	Surprisingly efficacious	None
Case 3	7	Cultural relevance	Provide more offerings to hold the emotions brought up by the music
Case 4	180	Cultural relevance	Fix the technological or audio issues
Case 5	96	Provided an acute escape	None

## Discussion

### Principal Findings

The goal of this study was to assess the feasibility of a digital music-based mindfulness intervention for Black Americans with elevated race-based anxiety. Overall, we found preliminary evidence that the intervention reduced state anxiety, even when accounting for the natural tendency for state anxiety to fall naturally over time. Additionally, participants provided high ratings for the feasibility and acceptability of the intervention across virtually all key metrics.

### Putative Mechanisms

#### Increased Mindfulness

Increased mindfulness is one clear potential mechanism that may underlie the results of this study. The intervention was designed to increase mindfulness and featured explicit mindfulness instructions tailored to the Black community. Additionally, the songs had mindfulness themes and practices woven into the lyrics. Furthermore, the acceptability score for “Improved Ability to Be Present” with thoughts and emotions received an average score of 90/100, providing preliminary evidence for this putative mechanism. Systematic reviews and meta-analyses have demonstrated that mindfulness-based interventions can lead to acute reductions in state anxiety [47-49]. Additionally, music-based interventions have shown preliminary evidence that they can elicit reductions in state anxiety through increases in mindfulness as well [29]. Although this is the most obvious potential mechanism for the present intervention, we did not directly test this mechanism within this study. Future inquiries should more directly assess the ability of this intervention to increase mindfulness.

#### Increased Self-Compassion

Self-compassion is another potential mechanism by which this intervention may reduce state anxiety. A central aim of the intervention was to increase self-compassion by including language that promoted self-acceptance and self-kindness. Meta-analyses examining self-compassion-related therapies (ie, interventions with an explicit goal of increasing self-compassion) have shown increasing self-compassion leads to reductions in anxiety and other psychiatric disorders [50,51]. Additionally, in a 2022 cross-sectional survey of Black, Indigenous, and People of Color (BIPOC) college students, a central aspect of self-compassion (ie, self-judgment) moderated and weakened the link between racial discrimination and anxiety symptoms [52]. Our study also found preliminary evidence of self-compassion as a putative mechanism, as the mean score for “Improved Relationship to Self” was (72/100). Despite this score being the lowest out of the feasibility and acceptability metrics, even this moderate score is noteworthy; one’s relationship to the self is likely entrenched and requires significant time and effort to change. Further investigation is needed to shed light on the ability of this intervention to promote self-compassion.

#### Cultural Resonance

Cultural resonance may be an additional mechanism to explain the efficacy of the intervention. Extensive meta-analyses and systematic reviews have shown culturally adapted mental health interventions to be significantly more effective than non-adapted interventions [53-57]. Additionally, interventions designed to target specific cultural groups can be up to 4 times more effective than nonadapted interventions [53]. Furthermore, mental health interventions tailored for the Black community have consistently shown substantial effectiveness [58-62]. In this study, 2 participants noted the Black cultural elements of the intervention as a core strength as well. Additionally, the mean score for the extent to which participants felt that the intervention was “Made for you” was 93 out of 100, providing additional evidence for this putative mechanism. Future research should explore cultural resonance as a mediator of this intervention for the Black community.

### Limitations

There are several notable limitations to this study. Although multiple-baseline designs are useful for initial preliminary inferences, their small sample sizes and within-subject comparisons limit the ability to decisively infer causality from one’s results. Furthermore, the open-label design is limited as well, as subject-expectancy effects may have contributed to our findings. Therefore, future trials should use well-powered randomized designs and between-subject controlled comparisons to further establish the efficacy of the intervention as well as potential mediators and moderators of intervention effects, the latter being of particular importance given the intervention was not equally effective for all participants in this study.

Next, the “Scan” package that we used to conduct our analyses has limitations as well. Specifically, the authors of this package note that the method they use to calculate confidence intervals for Tau-U analyses represents a conservative approach, meaning that the true confidence intervals for our results are likely narrower than reported. However, this limitation does not significantly hinder our study and, in fact, indicates that our results still reached statistical significance under more stringent conditions.

An additional limitation in our study is that for many of the participants, anxiety was steadily decreasing prior to the commencement of the intervention, raising questions about whether the intervention significantly impacted anxiety in our study. As previously mentioned in our methods, we anticipated and controlled for this fact in our study using Tau-U analysis, which allows one to incorporate any such trends into one’s comparisons of the intervention and baseline phases in a multiple baseline study. Nevertheless, future studies with larger sample sizes and more participants with stable baseline anxiety can further address this limitation.

Another potential limitation is that, in some cases, the intervention may be harmful or iatrogenic. For instance, anxiety spiked for 2 participants during the intervention, suggesting an iatrogenic effect. However, these increases in state anxiety may paradoxically have therapeutic utility as well, as they may represent the capacity of the intervention to facilitate the confronting of challenging, unprocessed emotions. For example, even though case 3 did not have statistically significant reductions in state anxiety, this participant indicated that the intervention indeed allowed her to confront difficult, unprocessed emotions of her own; she also provided the intervention with the highest possible scores for “Helpful for anxiety” (100/100) and “Likely to recommend” (100/100). Overall, this participant’s experience sheds light on the complexity of understanding whether the intervention is operating in a manner that is therapeutically challenging or explicitly harmful. Further research is needed to better parse this distinction.

Next, the study may have limited external validity. Participants in this study had previous mindfulness experience and used music for emotional support, meaning that our results may not generalize to individuals without experience with mindfulness or who do not listen to music as frequently. In addition, as we recruited participants from Prolific, a web-based study platform, participants within this study may differ from Black Americans with elevated race-based anxiety in real-world, non–web-based settings. Lastly, the sample included in this study was a nonclinical and noninpatient sample, meaning the potential utility of the intervention may be limited in more severe populations. Future trials should test the efficacy of the intervention in mindfulness-naive, non–web-based clinical populations.

### Future Directions

This study’s preliminary findings warrant further exploration. First, future studies should seek to replicate these findings in other multiple-baseline studies as well as in studies with larger sample sizes in order to confirm the efficacy of the intervention. Additionally, longer-term follow-up is needed to determine whether the intervention can elicit lasting reductions in anxiety.

Second, future studies can test the intervention with other racial minority groups. Although the intervention was tailored for the Black community and featured Black cultural elements, it used inclusive language to talk about identity and mindfulness, meaning that it may resonate with other racial communities. Future studies can therefore explore the potential efficacy of this intervention for non-Black minority populations.

Third, future investigations should examine the efficacy of specific tracks within the intervention, which may help clarify the therapeutic properties of each component of the intervention. The intervention consisted of poetry, guided meditations, and songs. Analyzing the efficacy of these individual tracks may shed further light on how the intervention works and for whom it may be most beneficial. Furthermore, such investigations can also illuminate whether individual tracks can serve as ultra-brief (ie, 5-minute) mental health interventions for the Black community as well.

Fourth, future studies should use participant feedback to update the intervention. In this study, 1 participant requested meditation instruction that included less jargon (case 1); another participant suggested including more poetry and metaphors in the artistic offerings (case 2). Future studies can incorporate this feedback into the intervention and test whether there are increases in therapeutic efficacy, feasibility, and acceptability as a result.

Fifth, future studies should examine potential moderators. As previously mentioned, all participants in this study were familiar with meditation and had used music for emotional support. Therefore, future studies can test these factors as potential moderators of our results as such investigations can shed light on the contexts and populations for whom this intervention is most helpful.

Finally, future studies can test the dissemination and implementation of the intervention. As the intervention is digital, this research team eventually plans to disseminate the intervention widely on the internet through music streaming platforms (eg, Spotify and Apple Music), offering free and low-cost avenues for accessing the intervention. Upon release, future naturalistic implementation studies can assess how this intervention impacts the mental health of the Black community, as Black Americans in real-world contexts can share their experiences with the intervention via web-based surveys. These individuals can also provide information about how they use the intervention (eg, in acute moments of anxiety, to practice mindfulness), providing us with a greater understanding of the utility of the intervention for the Black community. Implementation studies can assess scalability by gathering information on the number of individuals who access the intervention and the number of times the intervention is streamed on the internet. Overall, such studies can allow us to understand the real-world effectiveness and uptake of this therapy.

### Conclusions

This intervention demonstrated robust effects for reducing state anxiety and received strong feasibility and acceptability scores, suggesting that this intervention may offer a promising new avenue for addressing race-based anxiety for Black Americans. Future research is needed to replicate these preliminary results, assess mechanisms of change, examine efficacy in additional populations, and test the intervention in fully powered randomized trials. In sum, this study represents a promising first step in providing freely available and culturally relevant mental health support to the Black community.

## Appendix

Multimedia Appendix 1 Power analyses and individual Tau-U results.

## Abbreviations

CONSORT: Consolidated Standards of Reporting Trials
STAI-6: State-Trait Anxiety Inventory-6
