# Supplementary material for: A Digital Music-Based Mindfulness Intervention for Black Americans With Elevated Race-Based Anxiety: A Multiple-Baseline Pilot Study
Source: JMIR Form Res. 2023 Aug 16;7:e49284. doi: 10.2196/49284 (PMC10468709; doi:10.2196/49284)
Supplement: Multimedia Appendix 1 [file formative_v7i1e49284_app1.docx]

**Multimedia Appendix 1**

**Table S1.** Power analyses for 5 baseline periods and 12 intervention periods for 3 participants to detect a moderate intervention effect (d = 0.5) using overall Tau U analyses. Conducted using 500 monte-carlo simulations.

| Method | Power | Alpha Error | Alpha:Beta | Correct | p-value |
| --- | --- | --- | --- | --- | --- |
| Overall Tau-U Analyses | 84% | 4% | 1:4.1 | 89.9 | 0 |

**Table S2.** Individual Tau-U Results.

|  | Model | Tau | SE | CI lower | CI upper | z | P |
| --- | --- | --- | --- | --- | --- | --- | --- |
| **Case 1** | | | | | | | |
|  | A vs. B | –1.00 | 0.30 | –1.58 | –0.42 | –3.37 | <.001 |
|  | A vs. B - Trend A | –0.92 | 0.37 | –1.64 | –0.20 | –2.50 | <.05 |
|  | A vs. B + Trend B | –0.73 | 0.19 | –1.11 | –0.36 | –3.83 | <.001 |
|  | A vs. B + Trend B - Trend A | –0.62 | 0.17 | –0.96 | –0.28 | –3.60 | <.001 |
| **Case 2** | | | | | | | |
|  |  |  |  |  |  |  |  |
|  | A vs. B | –0.94 | 0.27 | –1.47 | –0.41 | –3.47 | <.001 |
|  | A vs. B - Trend A | –0.70 | 0.32 | –1.33 | –0.07 | –2.17 | <.05 |
|  | A vs. B + Trend B | –0.65 | 0.19 | –1.03 | –0.28 | –3.44 | <.001 |
|  | A vs. B + Trend B - Trend A | –0.44 | 0.16 | –0.75 | –0.12 | –2.69 | <.01 |
| **Case 3** | | | | | | | |
|  | A vs. B | –0.20 | 0.25 | –0.70 | 0.30 | –0.79 | .42 |
|  | A vs. B - Trend A | 0.12 | 0.30 | –0.46 | 0.70 | 0.39 | .69 |
|  | A vs. B + Trend B | –0.18 | 0.19 | –0.55 | 0.20 | –0.93 | .35 |
|  | A vs. B + Trend B - Trend A | 0.02 | 0.15 | –0.28 | 0.32 | 0.14 | .88 |
| **Case 4** | | | | | | | |
|  | A vs. B | –0.65 | 0.24 | –1.12 | –0.18 | –2.71 | <.01 |
|  | A vs. B - Trend A | –0.42 | 0.28 | –0.97 | 0.13 | –1.51 | .13 |
|  | A vs. B + Trend B | –0.43 | 0.19 | –0.81 | –0.06 | –2.26 | <.05 |
|  | A vs. B + Trend B - Trend A | –0.21 | 0.15 | –0.50 | 0.08 | –1.44 | .15 |
| **Case 5** | | | | | | | |
|  | A vs. B | –1.00 | 0.23 | –1.45 | –0.55 | –4.32 | <.001 |
|  | A vs. B - Trend A | –1.00 | 0.27 | –1.53 | –0.47 | –3.70 | <.001 |
|  | A vs. B + Trend B | –0.93 | 0.19 | –1.31 | –0.55 | –4.78 | <.001 |
|  | A vs. B + Trend B - Trend A | –0.67 | 0.14 | –0.94 | –0.39 | –4.78 | <.001 |
